# “Proud, brave, and tough”: women in the Canadian combat arms

**DOI:** 10.3389/fsoc.2024.1304075

**Published:** 2024-05-13

**Authors:** Emalie Hendel, Kate Hill MacEachern, Alma Haxhiu, Barbara T. Waruszynski

**Affiliations:** ^1^Director General Military Personnel Research and Analysis, Department of National Defence (DND), Ottawa, ON, Canada; ^2^Atlas Institute for Veterans and Families, Ottawa, ON, Canada; ^3^Military Personnel Generation Training Group, Canadian Armed Forces Borden, Ontario, ON, Canada

**Keywords:** combat arms, diversity, gender, inclusion, masculinized culture, military career, military culture, women

## Abstract

Canada’s defence policy, *Strong, Secure, Engaged*, emphasizes the importance of leveraging Canada’s diversity to strengthen the Canadian Armed Forces. Currently, women in the Canadian military are underrepresented across most elements and occupations, especially in the combat arms occupations, including among officers and non-commissioned personnel in combat units such as infantry, armored corps, artillery, and combat engineering. Research suggests that the benefits associated with the inclusion of women in combat arms occupations include an increase in collective intelligence, operational effectiveness, task cohesion, and diversity. This article explores the gender gap in the Canadian combat arms by examining the findings from two recent qualitative research studies on the perceptions of women in the Regular Force and Primary Reserve. The authors analyze female military personnel’s perceptions of women serving in the combat arms, and the ways to increase their inclusion in the military. The key findings reveal the following themes on women’s perceptions of servicewomen in the combat arms: great job for those who want it; challenging environment (e.g., working within a masculinized culture, necessary toughness, tokenism and the “pink list,” being treated differently, and family loyalty); unique challenges faced by women in combat roles; combat takes a toll on women’s mental and physical health; and benefits of women’s participation in multinational operations. The discussion highlights the need to increase diversity, equity, and inclusion, promote a culture change that fosters greater inclusion of women in the combat arms, and increase operational effectiveness through training and policies.

## 1 Introduction

Many nations continue to promote greater diversity, equity, and inclusion within their armed forces (Pendlebury, 2020). Whereas diversity recognizes the unique experiences, perspectives, and backgrounds of individuals (Department of National Defence, 2016), equity ensures that people from various groups have impartial and equitable access to opportunities and benefits (Government of Canada, 2023a). Inclusion is an active process of creating and changing the social environment so that individuals with varying identities feel welcomed, respected, and valued, culminating in a sense of belonging and engagement (Government of Canada, 2023a). Employing women in military occupations represents a top-down commitment to these principles on the part of the military organization (Davis et al., 2021). Male and female military personnel emphasize the importance of equality and the need to provide opportunities for women to advance operational effectiveness in the military (Harvey, 2019).

From a global perspective, the integration and representation of women in military organizations has been slowly increasing over the last 50 years (King, 2013). However, only two dozen nations allow women to participate in all military roles, including combat arms occupations (Goldstein, 2018; Percy, 2019 in MacKenzie and Gunaydin, 2022). Combat arms occupations are those in which troops participate in frontline military combat. These jobs include infantry, armored corps, artillery, and combat engineering positions, as well as air force and naval combat roles (Government of Canada, 2023b). In some countries, women have been actively participating in these occupations for decades (King, 2013, 2016). However, as will be discussed herein, the inclusion of women in combat roles is much more recent in other nations (Crowley and Sandhoff, 2017; Fieldhouse and O’Leary, 2023).

The inclusion of women into combat occupations highlights several challenges. There is a limited amount of research analyzing the various facets of integrating women into combat roles, and more specifically, how these would influence stereotypes, leadership, operational effectiveness, cohesion, and attitudes of members already in the combat arms. To better understand women’s experiences and perceptions in combat occupations, we must first recognize the factors that influence integration, the challenges of working in a masculinized culture, the impacts on operational effectiveness, and the attitudes toward women in the combat arms. As a result, the purpose of this paper is to examine these challenges through the perceptions of women serving in the military, including the combat arms.

### 1.1 Factors driving the integration of women in combat occupations

The history of women’s participation in military activities is blurred by societal norms, exclusionary laws and practices, and sensationalism (Soules, 2020). Nevertheless, their contributions to global military efforts have been present throughout history (see Carreiras, 2006 for a full review; see also Trisko Darden, 2015). Still, the formal integration of women into military occupations as full status began only a little over three decades ago (1989), including the participation of women in combat occupations (Canadian Human Rights Commission, 1989).

The early integration of women into combat roles came about following a Canadian Human Rights Tribunal ruling in 1989, where all occupations, including combat arms, except for submarine service (opened to women in 2001), were accessible to all women (Department of National Defence, 2018). The geopolitical pressures, military needs, and women’s push for equal rights also played fundamental roles in enabling women to pursue all occupations in the Canadian military. For example, recruitment difficulties, women’s widespread entry into the labor force, and political promises with regards to women’s rights and gender equality earned women in Sweden, Norway, and Denmark access to many military positions in the early 1970s (Ahlbäck et al., 2022). Shortly thereafter, women in these military organizations pushed for their right to advance, both horizontally and vertically, through the institutions. A decade later, Scandinavian women became the first in the world to officially be allowed to participate in combat arm roles (Ahlbäck et al., 2022).

In some countries, women have only been allowed into combat positions following specific legal actions and rulings of discrimination (King, 2013; Berezin Cohen and Netzer, 2023). For example, in the 1990s, after a woman was denied access to pilot examinations because of her gender, the High Court of Justice granted women in the Israel Defense Forces (IDF) equal rights to all military positions including combat duties (Berezin Cohen and Netzer, 2023). However, women in the Israel Defense Forces are still excluded from combat positions due to a high probability of engagement or being faced with extreme physical demands (Fieldhouse and O’Leary, 2023). Moreover, in 2000, after a German woman was denied access to a voluntary position which required the use of firearms, the European Court of Justice ruled that the exclusion of women from all military posts was not legally justified and therefore should not continue (Kümmel, 2002; King, 2013).

Women’s successful performance in combat roles also enabled them to access all military positions (Crowley and Sandhoff, 2017; MacKenzie and Gunaydin, 2022; Fieldhouse and O’Leary, 2023). For example, owing to their successes in Air Force combat roles since 1988, women in New Zealand were officially integrated into all combat occupations in the early 2000s (MacKenzie and Gunaydin, 2022). More recently, military needs in Iraq and Afghanistan required women to be placed into previously male roles in combat units. Women’s performance and the additional strengths they brought to these teams subsequently earned them official recognition and access to all combat roles in the United States and the United Kingdom (Crowley and Sandhoff, 2017; Fieldhouse and O’Leary, 2023).

#### 1.1.1 Challenges of working in a masculinized culture

Many arguments surrounding women’s inclusion in military occupations center around *stereotypes* about gender, which is defined as a social construct encapsulating various norm-related behaviors, physiological and psychological traits, cultural meanings, and one’s felt sense of identity (Government of Canada, 2023a; see also Lindqvist et al., 2021 for a discussion on gender in research). Since gender is viewed as a social construct, it brings about descriptive and prescriptive assumptions about one’s attitudes, characteristics, and behaviors (Crowley and Sandhoff, 2017; Stewart et al., 2021). As such, gender roles perpetuate gender inequality in many notable ways across all social sectors (see Stewart et al., 2021).

Historically, gender stereotypes have touted military service as being almost synonymous with masculinity and maleness (Goldstein, 2018; Deng et al., 2023). A masculinized culture can be perceived as an environment that perpetuates male ideologies, and social and cultural norms in a work environment, sometimes to the exclusion of women and other groups and communities (see Waruszynski et al., 2022). Combat roles are some of the most prestigious and exclusive occupations in any military organization, as they can be depicted as demonstrations of having ‘masculinized’ power over others (Goldstein, 2018). As Crowley and Sandhoff (2017) state, “the most masculine space is combat” (p. 222). As a result, women and combat are not compatible constructs. In essence, women are perceived as the givers of life, placing them in stark moral contrast to the role of a soldier who is trained to kill (see Peach, 1994). Beyond this, women are equated with needing protection, as they are depicted as weaker than men and more emotional, thereby manifesting contradicting gender norms and rules (McSally, 2011). If women in the military embrace their feminine characteristics, they are disregarded as being incapable of doing a soldier’s job. However, when women strive to achieve the honorable ideal of masculinity to be effective soldiers, they are taking on men’s roles and status as the dominant group (Connell and Messerschmidt, 2005; Goldstein, 2018; Van Gilder, 2019). To put it briefly, both attempting to change or to fit into military gender stereotypes challenges the notion that femininity is subordinate to masculinity. Thus, both strategies are refuted since they are disruptive to the culture of hegemonic masculinity which is present in most military organizations (Badaró, 2015; Crowley and Sandhoff, 2017; Goldstein, 2018).

Contending with the culture that has been created and maintained by the historically masculinized dominant group can be very challenging and can further generate multiple barriers for women wanting to succeed in military positions. Research has highlighted how servicewomen have had to portray themselves as “one of the boys” to fit in; reject notions of their femininity; work harder to prove themselves capable of doing the job; endure the negative perceptions of pregnancy and family responsibilities versus their capabilities and commitment to the armed forces; and challenge perceptions which attribute their success to their gender and not to their abilities (i.e., tokenism; Petite, 2008; King, 2016; Crowley and Sandhoff, 2017; Waruszynski et al., 2018, 2022; Davis et al., 2021).

A masculinized culture has also perpetuated sexual misconduct in the military. For example, the most recent survey of experiences of sexual misconduct in the CAF found that 67% of military members had witnessed or experienced a sexualized or discriminatory behavior in 2022 (Statistics Canada, 2023). While this represents a decrease in some behaviors, such as verbal or non-verbal communication, non-consensual physical contact and suggested sexual relations have increased since 2018 (see Statistics Canada, 2023). Although these behaviors were witnessed or experienced by members of differing genders, women were more likely than men to see, hear, or experience sexualized or discriminatory behavior (Statistics Canada, 2023). Most strikingly, 3.5% of members in the CAF reported having experienced sexual assault in 2022, and women were *three times* more likely to experience sexual assault than men (Statistics Canada, 2023). This represents an important increase over previous years in which less than 2% of military members indicated that they had experienced sexual assault; for example, rates of assault were 1.7% in 2017, and 1.6% in 2018 (Statistics Canada, 2023). Altogether, these examples show that women’s experiences in militaries around the world continue to be shaped by their gender, whether through experiences of discrimination and prejudice, career constraints, harassment and discrimination, or sexual violence, as examples (Badaró, 2015; Crowley and Sandhoff, 2017; Munshi and Pandey, 2017; Brown et al., 2021; Deng et al., 2023).

The challenges attributed to women working in a masculinized environment can be presented within a social identity theoretical framework, as proposed by Tajfel and Turner (1979; see also Tajfel, 1974). Social Identity Theory (SIT) examines the characteristics that describe people’s membership with groups (e.g., in-group versus out-group). In the research on women in the military, SIT helps to discern the social identities that women undergo as they try to belong to the membership of a group (e.g., women trying to belong to a traditionally masculinized group; Veldman et al., 2021). Women may start to behave and communicate in ways that enable them to have a greater sense of belonging to the predominant masculinized group. The focus is on belonging to a masculinized group and taking on male-like attitudes and behaviors, thus moving away from those women who display more feminine qualities and behaviors. As a result, women tend to “distance from other women as a way to fit in a masculine domain” (Veldman et al., 2021, p. 118). Social identity threats become pervasive, and some women may feel intimidated if they identify with other women within a military context. From a social identity perspective, Veldman et al. (2021) highlight that “self-group distancing is an individual mobility strategy to cope with a threatened identity” (p. 118). As a result, there is a need to challenge masculinized norms suggesting that masculine stereotypes equated with military effectiveness may have negative implications on cultural inclusion.

#### 1.1.2 Operational effectiveness

The question of operational effectiveness is often raised when it comes to women’s inclusion in military operations. Research highlights that personnel who do not display hegemonic traits such as physical strength, courage, or aggression, are seen to be putting military effectiveness at risk (Van Gilder, 2019). In fact, there is a stereotypical sentiment that women are physically inferior to men, which puts them at a disadvantage and at greater risk when they participate in combat activities (Munshi and Pandey, 2017). Yet, women already meet the physical and professional standards for military participation around the world. For example, the Canadian ‘Fitness for Operational Requirements of Canadian Armed Forces Employment’ (FORCE) evaluation fitness test and the standard for Universality of Service are the same for all prospective military members, regardless of gender or age (Canadian Forces Morale and Welfare Services, 2017). Another example focuses on the IDF where service in the military is mandatory for all citizens: there are no physical requirements for entry into the Israeli military service (Fieldhouse and O’Leary, 2023). Though these nations take different approaches to evaluating the physical fitness of prospective members, they both refute the premise that women are not physically suited to taking on military tasks.

When necessary, differences in physical strength, power, and military task performance between men and women can be reduced through resistance training (Kraemer et al., 2001). If need be, women can undergo training and adopt alternative methods of completing tasks, both of which give them greater opportunities to meet the challenges of the military and the combat arms (Waruszynski et al., 2022; Fieldhouse and O’Leary, 2023).

In brief, the resounding conclusions from the literature indicate that women are equally capable of gaining and demonstrating the capabilities and traits necessary to serve as effective soldiers. Thus, if women are fit to be soldiers and meet the criteria for deployment, then their gender should not deny their ability to contribute to operational effectiveness (Egnell, 2016). Unfortunately, negative attitudes attributed to women’s integration in the military, including the combat arms, continues to be perpetuated by a masculinized culture of gendered stereotypical roles and social norms that styme inclusion (Waruszynski et al., 2018; Van Gilder, 2019).

#### 1.1.3 Attitudes toward gender integration

As a result of the hegemonic masculinized culture of most military organizations, there is often a caveat associated with positive statements made about women in combat roles. When examining comments toward American women in combat roles, one online user highlighted, “as long as females pull their weight and do what needs to be done and not create a spectacle of themselves, the guys do not see a difference either” (Brownson, 2014, p. 773). Despite indicating support for women, what is said may imply a lack of trust in women’s abilities and the notion that women can potentially “make a spectacle of themselves” is derogatory and suggests that women hold an inferior role to men. These sentiments can become internalized, with servicewomen often condemning their own feminine traits, adopting stereotypically masculine characteristics, and holding women responsible for the shortcomings of men (Van Gilder, 2019).

Women also disproportionately face questions about the impact of their career decisions on their familial obligations. Since family responsibilities and roles, such as motherhood and caretaking, are traditionally feminine, women often find it difficult to balance these roles with their career pursuits (MacKenzie and Gunaydin, 2022). Often, where men are lauded for their service, women may be chastised for choosing an occupation that takes them away from their families (Waruszynski et al., 2018; Van Gilder, 2019). Even when they manage to find a balance between the two worlds, women are expected to care for their family’s post-deployment (Trisko Darden, 2015). These attitudes affect the recruitment and retention rates of women in many gender-integrated militaries around the world (Badaró, 2015; MacKenzie and Gunaydin, 2022). In non-integrated military organizations, combat roles are seen as posing a risk to traditional family structures, thereby excluding women from these positions (Soules, 2020). Altogether, instead of pushing toward a cultural shift, these issues work to uphold the masculinized culture of the military and continue to be used to justify the exclusion of women in certain roles, including combat arms.

### 1.2 Canadian combat arms

The CAF is made up of approximately 68,000 Regular Force members, 27,000 Reservists and 5,200 Rangers (Government of Canada, 2023b). These members are divided among three main branches: Canadian Army, Royal Canadian Navy, and Royal Canadian Air Force. Each branch contributes to the core missions outlined in Canada’s defence policy, *Strong, Secure, Engaged* (Department of National Defence, 2017a), including defending Canada and North America, contributing to NATO efforts and security abroad, responding to international and domestic disasters, and conducting search and rescue operations. The CAF works toward these goals through patrolling and monitoring activities, conducting peace-support and humanitarian operations, assisting civil authorities, and engaging in combat operations, the last of which ended in Afghanistan in 2011 (Department of National Defence, 2017a).

**Figure 1:**
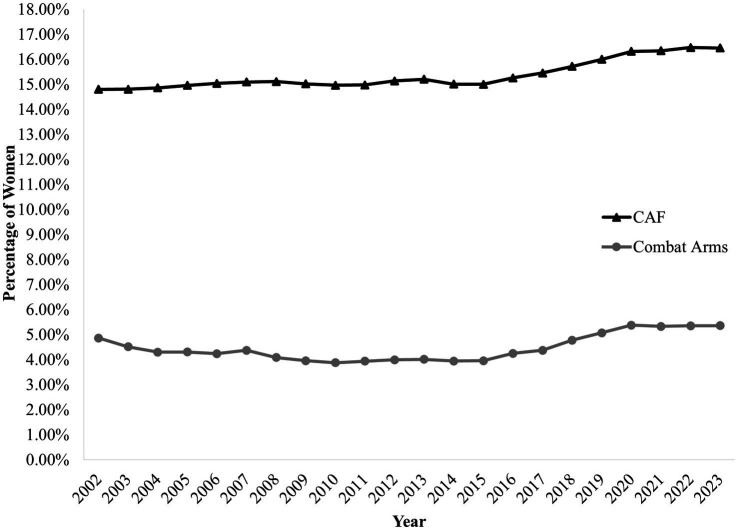
Representation of women in the CAF across all professions and in the combat arms from 2002 to 2023.

Women have been a part of the Canadian military for over a century (Davis, 1966). However, they were only granted access to the combat arms in 1989 after Canada’s Human Rights Commission Tribunal found their exclusion from these jobs to be discriminatory (King, 2013; Department of National Defence, 2018). Over the last 21 years, however, the representation of women in the CAF has increased by less than 2% (see Figure 1; Department of National Defence, 2023). At present, representation rates for women in the CAF sit around 16.5% (Department of National Defence, 2023) and far from the mandated 25.1% by 2026. In the combat arms, representation remains below 6% across the Regular Force and the Primary Reserve (Department of National Defence, 2023). Despite numerous recruitment and retention efforts, this number has not grown significantly over the last two decades (see Figure 1).

In summary, there are many challenges and attitudes which can contribute to the lack of women’s participation in combat occupations. Issues attributed to working in a masculinized environment (e.g., harassment and sexual assaults) demonstrate the challenges that women in the military face, including women serving in the Canadian combat arms.

### 1.3 Current study

As highlighted above, many of the complexities surrounding the inclusion of women in combat occupations stem from broader questions and perceptions about women in the military. The purpose of this article is to examine more closely Canadian servicewomen’s perceptions of women serving in the combat arms. The authors summarize the findings and recommendations given by women in the CAF Regular Force and Primary Reserve in response to the question, “What are your perceptions of women serving in the combat arms?” Responses broached several overarching themes, including job fit, working in a challenging environment, challenges unique to women, and involvement in multinational operations. Moreover, recommendations from participants highlighted ways to increase the recruitment and retention of women in the CAF. Overall, this journal article aims to clarify several issues that may need to be addressed to help increase the representation of women in the CAF and to promote the inclusion of women in the combat arms.

## 2 Methods

### 2.1 Participants

The participants included *N* = 503 female members of the CAF Regular Force (*n* = 335) and Primary Reserve (*n* = 168)^1^ who were recruited from bases across Canada. Participants represented a wide range of ages, military ranks, service elements, years of service, geographical regions, education levels, marital statuses, and languages. A complete breakdown of the participants’ demographics can be seen in Tables 1–3. The sample also included women who had participated in combat arms occupations.

**Table 1:** Age, highest level of completed education, and region of participants.

	Regular Force		Primary Reserve	
	*n*	% total sample	*n*	% total sample
Age (*n*=503)				
<25 years	24	4.8	42	8.3
25–34 years	123	24.5	53	10.5
35–44 years	130	25.8	35	7.0
45 years and over	58	11.5	38	7.6
Highest Level of Completed Education (*n*=494)				
High school diploma	81	16.1	21	4.2
College or CEGEP diploma	69	13.7	45	8.9
University certificate or diploma (below Bachelor’s level)	16	3.2	21	4.2
Bachelor’s degree	113	22.5	63	12.5
Graduate degree (e.g., Master’s or Doctorate)	52	10.3	13	2.6
Region (*n*=503)				
National Capitol Region (either Ontario or Québec)	14	2.8	13	2.6
Ontario (excluding NCR)	92	18.3	7	1.4
Québec (excluding NCR)	37	7.4	55	10.9
Atlantic Provinces	54	10.7	38	7.6
Prairie Provinces	91	18.1	32	6.4
British Columbia	47	9.3	23	4.6

The total number of cases for participants’ highest level of completed education does not add up to *N* = 503 because of missing demographic data. However, the percentage of the total sample is based on *N* = 503 participants for all variables.

**Table 2:** Military rank, element affiliation, and years of military service of participants.

		Regular Force		Primary Reserve	
		*n*	% total sample	*n*	% total sample
Military Rank (*n*=429)					
Junior NCM^a^	Private/Ordinary Seaman/Able Seaman/Master Corporal/Master Seaman	32	6.4	46	9.1
	Corporal/Leading Seaman	67	13.3	44	8.7
Senior NCM^a^	Sergeant/Petty Officer 2nd Class	30	6.0	13	2.6
	Warrant Officer/Petty Officer 1st Class	22	4.4	2	0.4
	Master Warrant Officer/Chief Petty Officer 2^nd^ Class, and Chief Warrant Officer/Chief Petty Officer 1st Class	14	2.8	8	1.6
Junior Officer	Second Lieutenant/Acting Sub-Lieutenant, Lieutenant/Sub-Lieutenant, and Captain/Lieutenant (N)	76	15.1	24	4.8
Senior Officer	Major/ Lieutenant-Commander, and Lieutenant-Colonel/Commander	43	8.5	8	1.6
Element Affiliation (*n*=493)					
Sea		83	16.5	44	8.7
Land		127	25.2	72	14.3
Air		62	12.3	21	4.2
Support		63	12.5	31	6.2
Years of Military Service (*n*=495)					
0–5 years		81	16.1	74	14.7
6–10 years		77	15.3	20	4.0
11–15 years		78	15.5	25	5.0
16–20 years		49	9.7	13	2.6
21–25 years		22	4.4	12	2.4
Over 25 years		26	5.2	18	3.6

The total number of cases for military rank, element affiliation, and years of military service do not add up to *N* = 503 because of missing demographic data. However, the percentage of the total sample is based on *N* = 503 participants for all variables.

^a^NCM refers to Non-Commissioned Members.

**Table 3:** First official language, marital status, and spouse in CAF.

		Regular Force		Primary Reserve	
		*n*	% total sample	*n*	% total sample
First Official Language (*n*=497)					
English		261	51.9	108	21.5
French		72	14.3	54	10.7
Both Official Languages		0	0.0	2	0.4
Marital Status (*n*=502)					
Single (never married)		87	17.3	76	15.1
Living common-law		41	8.2	25	5.0
Married		166	33.0	55	10.9
Separated/Divorced/Widowed		40	8.0	12	2.4
Spouse in CAF (*n*=168)					
Yes	Regular Force	*	*	58	11.5
	Primary Reserve	*	*	38	7.6
	Both	*	*	12	2.4
No		*	*	60	11.9

The total number of cases for first official language, marital status, and spouse in the CAF do not add up to *N* = 503 because of missing demographic data. However, the percentage of the total sample is based on *N* = 503 participants for all variables.

*This information was not available for the Regular Force sample.

### 2.2 Recruitment

Women currently employed with the Regular Force and Primary Reserve were invited to participate in the two studies by way of an invitation letter from the Commander Military Personnel Command (CMPC). Command Personnel Selection Officers and Base Personnel Selection Officers (BPSOs) assisted in communicating to potential participants the opportunity to take part in the qualitative research studies.

### 2.3 Data collection methods and procedure

For the current article, the findings are based on the results of two internal qualitative research studies on women’s experiences in the CAF which have been summarized and combined in this article. Two separate studies were conducted with servicewomen from the Regular Force and the Primary Reserve. Women in both studies were asked about why they joined the CAF, their experiences with recruitment, their perceptions of having a career in the military, and, for the purpose of the current paper, their perceptions of women in combat arms. Only the findings that examined servicewomen’s perceptions of women serving in the combat arms were summarized within this article. The complete findings are presented in other scientific reports and journals (see Waruszynski et al., 2018, 2022).

Participation in these studies was voluntary, and all participants were given informed consent forms to read and sign before taking part in the studies. With the help of BPSOs, researchers divided participants into various focus groups (*n* = 499) and individual interviews (*n* = 4). A total of 45 focus groups were conducted with participants from the Regular Force, and 24 focus groups and four individual interviews were conducted with members of the Primary Reserve. Approximately five to seven women took part in each focus group. Individual interviews took place when only one woman was available in a particular timeslot, or when a participant requested to speak to the researchers individually so that they need not share their thoughts and insights in a public forum. The focus groups and interviews were conducted in person and ranged between 60 and 120 minutes in duration. The questions were the same for the focus group and individual interview participants. Topics focused on one’s motivation to join the CAF, recruitment experiences, perceptions about their own careers in the CAF, and suggestions for attracting and recruiting more women. Specific to women in the combat arms, participants were asked, “What are your perceptions of women serving in the combat arms?” The focus groups and individual interviews were audio-recorded and transcribed before being coded and analyzed. This last question was analyzed and summarized to provide a better understanding of servicewomen’s perceptions of women serving in the combat arms.

### 2.4 Data analysis methods

In both qualitative studies, deductive and inductive approaches were used to code and analyze the data for the thematic content analysis. Both approaches allowed for an investigation of the themes found in the literature review and the themes that emerged from the data itself (see Proudfoot, 2023). Initially, the researchers conducted a literature review on women in the military. This allowed them to understand the context and to distinguish the key factors which could be used to code, organize, and analyze the data. A deductive approach also provided greater insights of the issues in the literature; however, the researchers needed to ensure that they were not introducing any biases (e.g., confirmation bias) into the analysis. Therefore, after transcribing the audio-recordings verbatim, the researchers employed an inductive approach by listening to the audio recordings of the focus groups and individual interviews. The researchers also developed a set of initial codes to establish the key categories found across the transcripts and summarized the main themes for a comprehensive understanding of servicewomen’s perceptions of women serving in the combat arms. This allowed the researchers to ensure that the data analysis was primarily guided by the overarching themes which were brought forward by the participants in this study. This dual approach allowed the researchers to understand the context better and further helped to ensure that the main themes were drawn from the focus groups and interviews.

Using MAXQDA software (version 18.0), the researchers then coded participants’ responses into common themes associated with the question on perceptions of women in the combat arms. Although the findings come from two qualitative studies, the goal for this article was to only analyze the responses from servicewomen’s perceptions of women serving in the combat arms. Responses from the focus groups and individual interviews were analyzed in the same manner since all participants responded to the same question.

To better understand how other intersecting personal identities (e.g., individual perceptions, attitudes, beliefs, and values and other factors that influence our perceptions about how we view ourselves in the world) and social identity factors (e.g., religion, ethnicity, race, gender, sexual orientation, etc.) could impact women’s experiences in the combat arms, the researchers employed a Gender-Based Analysis Plus (GBA+) approach (Government of Canada, 2023c). This tool uses an intersectional lens to assess and address inequalities within policies, programs, and initiatives (Department of National Defence, 2017a; Government of Canada, 2023c). This enabled the researchers to understand the participants’ different experiences, knowledge, and insights of serving in the Canadian military.

## 3 Findings

Overall, several key themes emerged on the participants’ perceptions of women serving in the combat arms, including: a great job for those who want it; challenging environment (i.e., working within a masculinized culture, necessary toughness, tokenism and the “Pink List”, being treated differently, family loyalty); combat not a place for women (i.e., combat takes a toll on women’s mental and physical health); and women’s involvement in multinational operations.

### 3.1 A great job for those who want it

Most of the participants viewed women in the combat arms positively, using words such as “proud,” “brave,” and “tough.” Many felt that it was a great career choice for a woman who wanted to be a part of that environment, and that having women in combat is important for the Canadian military and for operational effectiveness. It was acknowledged by most of the participants that it takes a special woman to succeed in the combat arms. As one participant stated, “There is a particular type of woman that will thrive in the combat arms and it’s not for every woman out there.” This statement seems to allude to the idea that some women possess specific characteristics which make them better suited to combat occupations. One participant explained the challenge,

For a woman that wants to be in the combat arms, you had better know how to be. And unless you are a competitor and you like being challenged all the time, and you … have to prove yourself all the time, because that’s what you are going to have to do unless you get to that line where you are one of them and then you are working and you are equal.

Within a SIT framework (Tajfel, 1974; Tajfel and Turner, 1979; Ellemers and Haslam, 2011), this type of statement clearly demonstrates the negotiation of various social identities, wherein women who belong to an ingroup from which they must distance themselves to become part of the better accepted ‘male-dominated’ outgroup. As one participant put it, women must “get to that line where you are one of *them*.” In other words, this participant is expressing the challenge that women who are interested in the combat arms must face to be included and accepted among their colleagues, who are predominantly men (see also Veldman et al., 2021 on self-group distancing).

In contrast, many participants spoke about how they were not interested in a combat role, but that they felt it was a great job for those who wanted it. A few participants pointed out that duties in combat roles can involve working in harsher conditions and these women stated that they preferred to be indoors where it is warm and dry. This was said to emphasize the fact that the combat arms was simply not an interesting career choice for many of these women. From a SIT perspective, these statements provide reasons for which women might move further toward their ingroup, rather than toward the male-dominated outgroup. Nonetheless, some findings with regards to those women who participate in combat occupations were generally spoken about as being positive. For example, physical fitness and the desire to be physically active, were attractive features of combat occupations. For many, it was gratifying to see what women could accomplish in terms of physicality and meeting the demands of training.

Overall, participants were clearly divided regarding the overarching theme that combat arms is not for everyone. For example, ‘toughness’ was a virtue of those who pursued combat roles, while others were intimidated by the need to be tough. Additionally, for some, combat arms occupations were a great way to break stereotypes and “shake off their femininity,” while others were wary of losing their female identity. While paradoxical, these findings demonstrate that women in the military and those already in the combat arms actively manage their social identities in various ways (Veldman et al., 2021). While women who are not in combat occupations may see those who are as possessing different traits and interests for themselves, they also recognize the challenges that women in the combat arms must face to be accepted. These perspectives reflect the active negotiation processes women in the military either observe or undertake to resolve being a woman in a male-dominated field.

### 3.2 Challenging environment

Participants, particularly those in combat roles, spoke about the benefit of being able to prove to oneself and others that one can perform challenging tasks. This was a major draw for women in the combat arms who wanted challenging work and thrived in that environment. Participants gave several reasons why the combat arms is viewed as a challenging environment, many of which were attributed to how women were perceived and treated in the units.

#### 3.2.1 Working within a masculinized culture

Women described the combat arms as being “macho” with “lots of testosterone.” This culture was thought to be challenging for women and a hard place to fit in because of the male dominant culture and the small number of women in combat units. A few participants felt that the combat environment would be even more difficult for women to succeed compared to other military occupations. Interestingly, for a few women in the combat arms who stated that they fit in well within their units, it was because they could be “one of the guys” or they found it easier to interact with male colleagues. This speaks to previous research where women found it necessary to take on masculine characteristics, like changes in physical appearance, such as cutting their hair short, taking on ‘masculine’ mannerisms, comportment, and postures (King, 2016), and in the ways they spoke about themselves and other service members (Van Gilder, 2019). Overall, the masculine culture permeated many of the issues and represents an important overarching theme in the perceptions of women in the combat arms.

#### 3.2.2 Necessary toughness

Women in combat roles felt that it was necessary to portray tough images. A physical toughness was essential, but so were having a strong personality and demeanor. However, having a tough image was also seen as a double-edged sword. To succeed, women felt that they needed to be tough; but, if they were too tough, they were often viewed negatively by their male counterparts. As one participant explained, “I’m not actually being snarky. I’m just wanting to do something and they are like no, no. And I’m like, yes. I’m asking you to do this thing rather firmly, so please go do it.” This was a frustrating challenge for many women when trying to establish respect and credibility among male colleagues and subordinates. One participant recounted the following situation, “I remember going through training and running a section attack and one of the guys in my section said, ‘When you start screaming, I tune it out.’”

Many women in the combat arms felt that they had to work harder to be accepted and prove their capabilities. Women felt they had to work to a higher standard before they were seen as capable by their male counterparts and needed to be better than everyone else. Once again, this reflects identity management strategies wherein women believe they must meet a certain standard before being valued and accepted by their male colleagues. Paradoxically, participants also described situations in which women were treated poorly when they outperformed their male colleagues. For example, one participant highlighted:

“So if you suck, they are going to drag you to the ground and frigging make sure you are out. If you are better than them, they are going to hate your guts because, my God, you make them look bad.”

In other words, if women outperform or exceed the standards laid out by their male counterparts, this poses a threat to the men’s own ingroup identity. Women who outperform these men challenge the latter’s worldview, and the extent to which they themselves meet their own group standards. In turn, men may act in such a way that reasserts their group’s superiority, such as punishing or dominating women (Ellemers and Haslam, 2011).

#### 3.2.3 Tokenism and the “Pink List”

Feeling like a token was a common theme among participants, as was the concern that women in leadership roles in the combat arms would be viewed as having achieved their status due to their gender and not their abilities. Participants referred to a “Pink List” that included names of women who could be promoted to certain positions, should senior leaders want to have a woman in that role. While the term “Pink List” originates from actions with the overall goal of ensuring fair access to career advancement opportunities for women such as reserving seats in staff college courses (McCristall and Baggaley, 2019), it has come to be used colloquially to include any action directed at career advancement for women. In terms of the results reported herein, participants’ references to a “Pink List” implies that the women on this list may not necessarily be fully qualified for the role but would get the opportunity as a quota requirement. This was thought to be a common perception of women’s roles in the combat arms – that women were sometimes seen as not having earned their place based on their knowledge, skills, and capabilities. For many, such a perception led to women having to work harder to prove that they had earned their role, and the additional difficulties in establishing credibility among subordinates when they were placed in positions of leadership over men. For example, one participant stated,

You do not want to go somewhere on a pink slip. You want to go based on merit. You want to be recognized because you are a good soldier, not because you are male, because you are female. You just want to be part of that team, and you want to be part of that cohesion.

#### 3.2.4 Being treated differently

Several women talked about the challenge of differential treatment from male colleagues. It was frustrating for some women in the combat arms to be asked if they needed help in carrying something heavy, for example. A few women also described situations in which military male personnel commented on women dressed in their military uniforms. For example, one participant reported being told by a male colleague, “You look so sweet.”

A few women also addressed concerns when interacting with male colleagues and the perception that friendly interactions were misconstrued. Participants suggested that women had to act a certain way to avoid being labeled in a negative manner. A few participants recalled giving advice to new members not to date anyone in their unit to avoid a negative reputation. In certain situations (e.g., while deployed), this became very isolating and lonely for women. Much of this was, again, attributed to the low number of women in the combat arms. In addition, there were some women who expressed frustration with reconciling their identity, whether having to pretend to be a different person at work or being reduced to their feminine identity. For example, one participant stated,

Because there are so few of us and there’s so many of them that even if you work alongside, you are someone’s wife, you are someone’s girlfriend. Like I’m not, you know, the logistics officer, I’m so-and-so’s wife.

#### 3.2.5 Family loyalty

Having to choose between having a family or a career was a common reason for why women did not pursue a job in the combat arms. As combat trades are demanding regarding time devoted to training exercises and deployments, many women felt that they could not comfortably care for their children and maintain a high level of commitment to their jobs. For those who were able to have a family and pursue a combat trade, it was often because they had a supportive spouse who was willing to take on more duties at home.

Women who were in the combat arms spoke about how family and friends had questioned them about their decision to pursue a combat arms occupation. Their loyalty to their family and ability as a mother were questioned. As one participant stated, “I had somebody ask me, ‘Don’t you love your children?’” Many women whose husbands were also in the military felt this was a frustrating double standard as their husbands were never asked these questions, nor were they assumed to be neglecting their family responsibilities when they went to work. Along this same line, another participant described an interaction with a fellow military member,

And I, this year, had a senior person tell me that I should just quit my job and raise my kids because it’s the best thing I could do for my children and my husband makes good money. We make the same money!

As a result, it can be perceived that a woman may not be acting in the best interest of her family and that she should be at home with her children.

#### 3.2.6 Unique challenges faced by women in combat roles

There were a few women who felt that women did not have a place in combat roles. For some, it was simply that they viewed women as not physically capable of performing the duties associated with combat roles. Women were sometimes viewed as too weak, both mentally and physically, to endure the conditions and expectations of the combat arms professions. Many participants questioned why a woman would even want to be part of that environment.

Just as in the study by Van Gilder (2019), a few participants raised concerns about men instinctively protecting women and how this may place units and fellow military members at risk. As one participant stated,

They [women] have the opportunity to do it, do they belong there? That’s where I…struggle with that. I do not think they belong there. Only because man’s perception is always to protect a woman, and what are they going to be doing differently if they have a female with them on some mission somewhere?

In addition, placing women within a large group of men was seen as dangerous by some participants, as there was the potential for sexual assaults from fellow soldiers. It was felt by a few participants from the Primary Reserve that the combat culture had changed because of women and the camaraderie that had once existed within all-male units was no longer present. This was viewed as a negative outcome of women’s integration and a detriment to effective military operations.

#### 3.2.7 Takes a toll on women’s mental and physical health

Some women believed that the physical requirements and demands of combat occupations were greater than those needed for other military roles. These participants felt that they could not meet the additional level of training and fitness that would be required for combat. However, participants who worked in combat trades spoke about this misperception and stated that the physical fitness standards were straight-forward and manageable once engaged in the right training.

Several women spoke about the toll that combat occupations take on a woman’s mental and physical health. For women in the combat arms, the physical breakdown of their bodies was challenging and spoke to the demanding conditions in combat roles. Many participants believed that a woman could not stay in a combat occupation for an extended period because of the toll it takes on a woman’s body. The most common example was pain and mobility issues related to knee and back injuries. While injuries stemming from military service including the combat arms are not solely experienced by women, the worry that occupations within the combat arms pose a greater physical risk to women than to men is not unfounded. In fact, from 2014 to 2017, musculoskeletal injuries resulted in more releases from military service for women than for men (Serré, 2018, 2019).

Coupled with the belief that women in the combat arms need to be mentally tough was the perception that the combat arms took a toll on women’s mental well-being. Women need to be resilient because it is a mentally challenging environment. Participants alluded to the culture, including the jokes and comments that they must endure. This also encompasses other issues that were raised, including having to continually work harder and prove oneself capable, or to have to remind fellow service members that servicewomen are qualified for their positions.

### 3.3 Women’s involvement in multinational operations

Servicewomen expressed two opposing perspectives when discussing women’s involvement in international missions. Some participants felt that women were essential to international missions and that there were certain roles that only women could fill. While these findings echo those of Egnell (2016), who clearly outlines the benefits of incorporating women into international peacekeeping operations, other results refute these suggestions. For example, a few participants felt that it was futile to send women to work alongside men in communities where women are not viewed as equals.

Again, these results highlight the complexities surrounding women’s integration into various military operations. On the one hand, some participants recognized and expounded the benefits that women could bring to multinational operations. This finding mirrors the goals and objectives outlined within the UN’s Women, Peace, and Security agenda. On the other hand, other participants viewed these efforts as being fruitless and ineffective, since other barriers exist which prohibit women from providing effective service while on operations.

Interestingly, while participants reported safety issues with respect to their involvement in multinational operations such as a fear of being at a higher risk of sexual assault, other challenges and negative impacts have also been reported within the literature. For example, Davis et al. (2021) highlight that because of the limited number of women in UN operations, any strategy that tries to reach a deployment gender quota could mean that the same soldiers are deployed time and again. Additionally, involving women in certain multinational operations could perpetuate gender stereotypes if these soldiers are only seen as filling a quota, and not as qualified servicemembers for each mission (Phoenix Strategic Perspectives, 2021). Therefore, the findings reported here are not unique to the current study; rather, similar beliefs and fears exist among women in militaries on a global level.

## 4 Discussion

This article examined the perceptions of women in the Regular Force and the Primary Reserve, specifically looking at women serving in the combat arms. Salient themes, such as diversity and opportunity, a masculinized military culture, and women’s contributions to the combat arms, were highlighted by the participants. For example, participants spoke about the combat arms as being a good job for women who possess certain characteristics, and the challenges of working within a masculinized culture where they may not feel included or treated fairly. A double standard highlights the expectations surrounding women’s roles in the family versus in the military. Some participants expressed the view that combat was not an environment in which women should work, and many were concerned with the toll of the combat arms on women’s mental and physical health. Based on the research findings and literature review, we now examine the findings with a focus on increasing diversity, equity, and inclusion by creating opportunities for women, enabling culture change, and increasing operational effectiveness through advancing policies, gender mainstreaming, strategies, and programs.

### 4.1 Increasing diversity, equity, and inclusion to create opportunities

Diversity, equity, and inclusion are fundamental principles which are intended to enhance the functioning of the CAF. Although most participants were adamant that the combat arms is not for everyone, their responses seem to indicate that women who pursue a career in the combat arms may share certain characteristics. These include having a desire to engage in physical fitness and the need to challenge oneself. This echoes Waruszynski et al. (2018, 2022) who found that participants perceived military occupations as being some of the only jobs which encourage and develop physical fitness. In addition, participants in the combat arms spoke about the enjoyment they got from challenging themselves and seeing what they could accomplish.

To increase opportunities for people of various personal and social identities, including women, the shared characteristics could be used to inform recruitment strategies and to determine how someone might fare in a combat situation. For example, women who engage in physical challenges, such as the Spartan races, may be interested in the combat arms. Having servicewomen participate in these events or having recruiters at the events may be a way to connect with women who possess an interest in physical fitness and the need to challenge oneself. Other innovative recruiting methods, like the Women in Force Program (Department of National Defence, 2017b), provide opportunities for women to “try-out” the CAF through exercises and workshops, giving them a practical understanding of what military careers entail. Spending a few days trying out the job is much less intimidating for women who may feel uncertain about the physical aspects of the job.

### 4.2 Culture change in the combat arms

It is clear from the participants’ responses that culture change is needed in the combat arms. This is no different than what has been suggested from female participants in general (see Goldstein, 2018; Waruszynski et al., 2018; Pendlebury, 2020; Waruszynski and MacEachern, 2021a; Fieldhouse and O’Leary, 2023). Numerous societal expectations based on sustained perceptions of hegemonic masculinity can place additional strain on women’s perceptions of themselves and their place in the military. This is especially relevant in occupations which may be closely tied to the stereotypical warrior image, such as careers in the combat arms (Crowley and Sandhoff, 2017; Waruszynski and MacEachern, 2019).

#### 4.2.1 Stereotypes and unconscious bias

Continued education about stereotypes and unconscious bias will be a key part of changing attitudes and behaviors toward women in the military. Women can perform duties just as well as men and should not have to continually prove themselves capable (Cawkill et al., 2009; Eichler, 2013; King, 2013; Crowley and Sandhoff, 2017). For example, participants in this work felt that they had to work harder to prove that they belonged in combat roles, lest they be subjected to humiliation and discriminatory comments. Likewise, women in the CAF should be treated in the same manner as their male counterparts when it comes to job performance. When women in this study did achieve the same performance standards as men or when they outperformed their male counterparts, they recounted being subjected to additional jokes and bullying. Despite their merits as soldiers, these findings clearly demonstrate the biased treatment of women in the military. However, this cycle of treating women differently only serves to reinforce and proliferate negative stereotypes and biases (Waruszynski et al., 2018).

Due to the masculinized culture, women in the military often choose to take on various masculine characteristics to feel included and respected (see also King, 2016; Waruszynski et al., 2018; Van Gilder, 2019). For example, our participants described “shaking off their femininity” and becoming “one of the guys” to fit into the predominantly male environment of the military and the combat arms. Attempting to amplify masculine characteristics can create a dichotomy in their identities and gender expressions, where a more “masculine” self exists within the armed forces, but feminine traits can be displayed in women’s social and personal lives (Badaró, 2015). Although men are also subject to the culture of hegemonic masculinity within most militaries, their expression of dominant masculine traits and their work toward achieving the persona of the “ideal soldier” allow them to be part of their in-group. In contrast, as participants’ responses demonstrate, even when women display these characteristics, they still face negative repercussions such as harassment, discreditation, exclusion, bullying, discrimination, and sexual violence (see also Crowley and Sandhoff, 2017; Munshi and Pandey, 2017; Pendlebury, 2020; Brown et al., 2021; Deng et al., 2023). As our participants described, some women are harassed, sexualized, or reduced to their feminine attributes even when they make attempts to fit into the dominant group. Even women in positions of leadership within highly masculinized environments, such as the military, can perpetuate existing gender stereotypes through micro-aggressions toward persons with the same gender identity (Derks et al., 2016). It is clear, therefore, that the culture of hegemonic masculinity is pervasive and shapes women’s military experiences at all levels.

Some participants alluded to women’s inclusion within combat arms occupations as representing a token and being placed on the “Pink List.” Put simply, it was the belief of some participants that women may be included in combat roles in some small numbers to fill gender quotas and to reach the bare minimum standard of gender integration. These comments refer to the concept of tokenism, where the underrepresentation of women within certain roles makes them more visible and subjects them to greater performance pressures (Perez and Strizhko, 2018). In other words, women in the combat arms may not be seen as full members who have earned their rankings, but as tokens of inclusion. This concept is a double-edged sword for women. On the one hand, they may receive special, preferential treatment and attention since they are a minority within a much larger group. On the other hand, their visibility can increase performance pressures and judgments of their work (Brookshire, 2017). This increased pressure and scrutiny can lead to isolation, micro-aggressions, and negative health and well-being outcomes (Perez and Strizhko, 2018).

In moving forward, it is important for the CAF to dismantle the hegemonic masculinity found within its culture and find a place for femininity and other gender expressions (Badaró, 2015; Tait, 2020; Phoenix Strategic Perspectives, 2021). This can be achieved by challenging gender stereotypes, by promoting the equitable treatment of women, and by recognizing and valuing the unique and diverse capabilities that women bring to the organization (Badaró, 2015; Van Gilder, 2019; Waruszynski and MacEachern, 2019; Tait, 2020; Waruszynski et al., 2022). If militaries continue to integrate women into the existing masculinized culture without allowing for a cultural shift, they risk losing the opportunities and benefits associated with gender-diversity (Davis et al., 2021).

#### 4.2.2 Respect and inclusion

The military possesses a cultural identity that is based on military service customs, traditions, practices, and values that encourage a team-oriented spirit and a sense of belonging; yet not all military personnel feel that they are part of an inclusive culture. For example, the research on women in the CAF highlight that although upholding respect and inclusion are key characteristics for a thriving military culture, many servicewomen feel the need to take on different strategies that would enable them to feel respected as capable soldiers and included as part of the military culture (Waruszynski et al., 2022). For instance, some servicewomen in the combat arms felt that they needed to work twice as hard to be accepted as soldiers; while others took on more male-oriented traits to fit into the predominantly masculinized culture (Waruszynski et al., 2022).

Moreover, based on previous research, the lack of respect toward women in the military is of grave consequence when it comes to gender-based issues and sexual misconduct. For example, women in the CAF are four times more likely to be sexually assaulted than men, and twice as likely to be subjected to sexualized or discriminatory behavior (Cotter, 2016). This includes sexual jokes and comments, unwelcome physical contact, offering work-related benefits in exchange for sexual activities, mistreatment, and exclusion due to gender or sexual orientation (see Cotter, 2016; Waruszynski and MacEachern, 2019). These types of experiences can affect a person’s career, health, and mental health (Watkins et al., 2017; Eichler, 2021). Since there is an even greater risk of sexual assault on women during deployments (Watkins et al., 2017), respect is especially critical for the safety of women in occupations such as the combat arms. Some participants worried that being in such occupations, and therefore being surrounded by large groups of men, could increase their risk of sexual violence. Still, it is important to note that sexual misconduct and discrimination based on gender or sex can occur at any point in time, and it is not limited to women working in the combat arms or to women in general (see *Heyder Beattie Class Action*, Department of National Defence, 2021). Nonetheless, sexual assault is more prevalent among members of the military who identify as women, who are young, Indigenous, have a disability, or who are not heterosexual (Statistics Canada, 2023).

Based on the current statistics (see Statistics Canada, 2023 for the latest figures), it will be important to reexamine the current thinking and the lack of understanding of the current policies and programs designed to eliminate sexual misconduct in the Canadian military. Therefore, respect and inclusion must be emphasized throughout all public communications and internal CAF messaging, policies, and systems (Waruszynski and MacEachern, 2021b). Further, the need for respect must be consistently promoted in leadership at the most senior level and across all personnel to help combat sexual misconduct, harassment, and discrimination across the armed forces.

### 4.3 Increasing operational effectiveness through training and policies

#### 4.3.1 Physical fitness and mental health

Many participants spoke about the challenges related to physical fitness standards. Physical fitness standards in the combat arms were perceived as a barrier to women’s participation in combat occupations. Although entry level fitness standards are the same as in other military occupations, participants were apprehensive about the additional training and fitness expectations of the combat arms. Many participants felt they could not achieve the standards necessary to perform combat roles. Still, as responses from participants who were employed in the combat arms demonstrate, this is a common misconception. In actuality, the participants in combat trades felt that the physical standards of combat were attainable once one was engaged in the correct training regimens. Findings from the global literature suggest the same thing: once women follow proper training programs, they can meet the physical standards and perform the tasks necessary to combat trades just as well as their male peers (Kraemer et al., 2001; Waruszynski et al., 2022; Fieldhouse and O’Leary, 2023). Even in militaries where physical fitness standards for entry into military service differ based on gender, such as in the United States, there has been a concession that there is no link between these tests and military occupations (Do and Samuels, 2021). So, despite the misperception that the combat arms trades require more physical fitness and are therefore unattainable, women have proven themselves to be very successful in these occupations (Trisko Darden, 2015).

It was also commonly stated that women’s bodies were different from men’s and performing the same duties had different physical impacts for women (see also Davis et al., 2021). Many participants were concerned about how their bodies broke down, including knee and back injuries. Nonetheless, proper training to support areas prone to injury may help prevent further injuries or minimize the impact of an injury (Kraemer et al., 2001). Ultimately, understanding how best to train a woman’s body may be an important part of retaining women in combat positions. Additionally, educating women about the physical fitness standards and standardized evaluations within the CAF could help to resolve this perceived barrier (see Waruszynski et al., 2022).

As for mental health, women in the current research studies expressed the need to be mentally prepared for any potential forms of discrimination (including discriminatory jokes), comments undermining their capabilities, and other forms of harassment. Gender-based harassment and discrimination have been associated with an increased risk of mental health disorders and suicidality (Brown et al., 2021). Moreover, military sexual trauma puts servicewomen and veterans at an even greater risk of suicide (Brown et al., 2021). Although these experiences do not just occur in combat occupations, the deep-rooted cultural issues of most military organizations may increase the incidence of these outcomes for women in the combat arms.

#### 4.3.2 Family-friendly policies

Attempting to balance family commitments and a combat occupation can be very challenging. In moving forward, the CAF must continue to adopt family-friendly policies and look at additional ways to assist servicemembers with family responsibilities. The family-work life balance has been detailed in many publications on women in the military (e.g., Badaró, 2015; Waruszynski et al., 2018; Waruszynski and MacEachern, 2021a,b) and speaks to a major barrier for women in military occupations. Women in the CAF Regular Force identified this as a concern (Waruszynski et al., 2018) and the ability to balance family and work commitments was a major reason why women pursued jobs with the Primary Reserve (Waruszynski and MacEachern, 2021b). Increasing family-friendly and gender-inclusive policies will help all members of the CAF but may be particularly beneficial to women in the military, especially those who are in combat arms trades (see Davis et al., 2021).

#### 4.3.3 Gender mainstreaming

Gender mainstreaming is the routine process of considering gender in public policy development, programs, activities, and initiatives (Department of National Defence, 2017a). The CAF has undertaken the objective of becoming a leader in gender mainstreaming by fully implementing the Women, Peace and Security agenda laid out by the United Nations (UN; Department of National Defence, 2017a). While their work is not and should not be limited to fulfilling gender-specific roles (see Egnell, 2016), women in the combat arms already contribute to the contemporary objectives of this agenda such as protecting human rights and supporting vulnerable and marginalized populations (Department of National Defence, 2017a). For example, servicewomen in Iraq and Afghanistan enhanced their teams’ situational awareness and access to information by communicating with local women who were prohibited from speaking to men (O’Connor, 2010; Egnell, 2016; Davis et al., 2021).

Women can also help to inform their teams about gender perspectives; address the specific needs of other servicewomen (e.g., mentoring female cadets, interviewing survivors of gender-based and sexual violence, etc.); serve as role models for women and girls in local communities; and identify the manifestation of inequalities in decision-making and power dynamics, both within the organization and on the ground (Egnell, 2016). Additionally, women have shown exceptional performance in ground combat and infantry (Cawkill et al., 2009; Eichler, 2013; King, 2013). Ultimately, women belong in the combat arms, both as soldiers engaged in combat and war fighting, and as peacekeepers. Nonetheless, despite these important contributions to various operations, women who are already serving in the combat arms continue to be questioned about their abilities, motives, and belonging within the organization (Waruszynski et al., 2022). Even some participants in the current study believed that the barriers faced by women in the combat arms made their service ineffective.

Increasing the number of women who participate in all aspects and at all levels of the military, including the combat arms, could become cyclical. For instance, individuals with friends or family in the military are those most likely to join the armed forces (Anderson, 2018; United Kingdom Parliament, 2019; Waruszynski and MacEachern, 2021a,b; Waruszynski et al., 2022). Increasing the number of servicewomen in the CAF means that more Canadians will have female role models to emulate, and more women will have opportunities to be promoted through the ranks. In fact, leadership is of utmost importance for organizational culture change and productivity, and increasing the number of women in leadership positions has been shown to reduce the use of gender-stereotyped language within an organization (Lawson et al., 2022).

It has been theorized that increasing the presence of women in combat roles would have a great impact on the operational effectiveness, policies, and culture of the armed forces (see Haring, 2013; Davis et al., 2021). However, a recent report by MacKenzie and Gunaydin (2022) found that although the Canadian and New Zealand militaries lifted their combat exclusions for women decades ago, the enrolment and retention of women into their armed forces has not greatly increased. Further, most women were only promoted into middle ranks, and very few attained higher leadership positions. In sum, opening combat roles to women was a change toward recognizing their contributions and capacities as effective soldiers. That said, the representation of women in these roles remains low, and has not yet brought about any significant cultural shift. At the level of the organization, the CAF has not made sufficient cultural changes to address the lived experiences of marginalized minorities or encourage their participation in combat (Phoenix Strategic Perspectives, 2021).

## 5 Limitations and future research

The largest limitation in the current study was the small number of women who were currently employed in combat occupations in our final combined sample. The very low representation of women in combat arms within the CAF would have resulted in a very small, non-representative sample even if we had been able to recruit all the women in combat occupations in the military. Still, our intent was not to gather a representative sample as in quantitative research. Rather, we aimed to build on the rich lived experiences of women serving in the CAF. The findings within this article can help to inform a future quantitative study which could examine the specific factors attributed to women working in the combat arms, both nationally and internationally.

Further, there is still a need to better understand the lived experiences of women in the combat arms and the reasons that other women would or would not join this profession. Nonetheless, our work centered only around women in the Regular Force and the Primary Reserve of Canada’s military. It would be of great interest to understand the perspectives of women who come from different nations and to make comparisons on the lived experiences and perceptions of women serving in the combat arms. For example, one recent study in Sweden investigated women’s and men’s perspectives on women in military occupations, including combat (see Linehagen, 2023). Unfortunately, despite Sweden’s long history of gender integration within the military and societal efforts toward gender-equality, the conclusions of this research reiterate our own findings. In brief, they point to a male-dominated organizational culture that is resistant to change (Linehagen, 2023). These similar findings lead us to believe that a broader global perspective should be sought. Importantly, this should be done through an intersectional lens to understand the perspectives from military members with diverse personal and social identities.

The need to address intersectionality within the CAF is undeniable. Factors such as sex, gender, sexuality, race, ethnicity, and Indigeneity, mental or physical disability, socioeconomic status, and age can intersect and create a set of experiences that are greater than the sum of the individual components (Crenshaw, 1989; George, 2020; Eichler, 2021; Eichler et al., 2021). As Eichler et al. (2021) state, in the military, “Layers of disadvantage may arise, for example, as a result of intersecting experiences of rank, trade, race, and gender” (p. 145). In the current study, disadvantages included inequitable treatment, health and mental health issues, and sexual harassment (see also Gill and Febbraro, 2013; Eichler, 2016; George, 2020; Eichler et al., 2021). Intersectionality should be addressed when recruiting and selecting new personnel, promoting members, conducting research, and creating or analyzing policies, strategies, and programs to enable greater inclusion (see Eichler et al., 2021). For example, using tools such as GBA+ can allow researchers to examine intersecting identity factors and their impacts on lived experiences, especially for underrepresented populations such as women in the combat arms (Eichler et al., 2021).

Finally, there is a need to create a framework and conceptual model through which to foresee and address challenges related to women’s participation in the combat arms. This framework and model could be used to reduce barriers to participation and to increase the recruitment of women into combat roles around the world.

## 6 Conclusion

This article aimed to illustrate the perceptions that current servicewomen have of women serving in the combat arms. The responses brought to light numerous perceptions and attitudes, including many challenges that are consistent with the international literature on servicewomen serving in the military, such as the embodiment of “masculine” characteristics (Badaró, 2015; Crowley and Sandhoff, 2017; Goldstein, 2018), being treated differently due to one’s gender (Petite, 2008; King, 2016; Crowley and Sandhoff, 2017; Waruszynski et al., 2018, 2022), and additional pressures due to societal expectations of family roles (Trisko Darden, 2015; Soules, 2020; MacKenzie and Gunaydin, 2022). Positive perceptions about women in the combat arms included the importance of the diverse knowledge and expertise that they bring to combat operations, including their capabilities as combat soldiers. However, a few serious drawbacks and barriers were also discussed. Many of these centered on the masculinized culture of the military, and specifically, the culture of the combat arms. The responses provide useful information as to what drives a woman to pursue these occupations, and what changes are required to make the combat trades more accepting and inclusive.

Based on the overarching research findings and literature review, we discussed the findings with a focus on increasing diversity, equity, and inclusion by creating opportunities for women, enabling culture change, and increasing operational effectiveness by advancing policies, gender mainstreaming, strategies, and programs. Overall, many of the findings presented a duality in the perceptions of women in the combat arms. While some participants had negative perceptions of women serving in the combat arms, others reported positive opinions and felt that women contributed significantly to combat-related missions and operations. This speaks to the complex nature of the perceptions of women serving in the combat arms.

Following from these findings, several recommendations were put forward to enable a greater culture change. First, the characteristics shared by many women who already occupy combat roles should inform recruitment and retention strategies. Furthermore, biases need to be addressed, inequalities challenged, intersecting identities acknowledged, and respect promoted throughout the combat arms. It is also important to train women in such a manner that respects their different physiological capabilities and protects them from injury, while allowing them to attain the same level of fitness and to perform the same duties as their male colleagues. Additionally, specific policies and programs should be put in place to protect and support women’s mental health, since they are more at risk of severe mental health issues and suicidality (Brown et al., 2021). Finally, family-friendly policies need to continue to be adopted, since women are disproportionately impacted by family-related responsibilities. Ultimately, implementing these organizational culture changes and promoting innovative recruitment and selection strategies will make the combat arms stronger, more diverse, equitable, and inclusive, and a top-of-mind professional career choice for women serving in the Canadian military and around the world.

## Data availability statement

The datasets presented in this article are qualitative and cannot be accessible outside the DGMPRA research team due to confidentiality and informed consent issues. Any questions regarding the data should be directed to barbara.waruszynski@forces.gc.ca.

## Ethics statement

The studies involving humans were approved by the Social Science Research Review Board (DGMPRA/DND). The studies were conducted in accordance with the local legislation and institutional requirements. The participants provided their written informed consent to participate in both studies.

## Author contributions

EH: Conceptualization, Writing – original draft, Writing – review & editing. KM: Conceptualization, Writing – original draft, Formal analysis. AH: Conceptualization, Writing – original draft. BW: Conceptualization, Data curation, Formal analysis, Investigation, Methodology, Project administration, Supervision, Validation, Visualization, Writing – review & chief editing.

## Footnotes

^1^Two female participants were from the Regular Force but provided suggestions on how to improve the recruitment of women in the Primary Reserve.
